# Are Uterine Manipulators Harmful in Minimally Invasive Endometrial Cancer Surgery? A Retrospective Cohort Study †

**DOI:** 10.3390/cancers17243906

**Published:** 2025-12-06

**Authors:** Maxime Côté, Marie-Claude Renaud, Alexandra Sebastianelli, Jean Grégoire, Ève-Lyne Langlais, Narcisse Singbo, Marie Plante

**Affiliations:** 1 Division of Gynecologic Oncology, CHU de Québec-Université Laval, Québec, QC G1R 2J6, Canada; 2 Clinical Research Center, CHU de Québec-Université Laval, Québec, QC G1V 4W6, Canada

**Keywords:** endometrial cancer, low-risk disease, uterine manipulator, minimally invasive surgery, oncological outcome

## Abstract

Objective: The objective of our study was to assess the oncological safety of uterine manipulators (UMs) in apparent early-stage (FIGO I-II 2009) endometrial cancer treated by minimally invasive surgery (MIS). Methods: Our single-center retrospective study includes patients who underwent endometrial cancer surgery for apparent early-stage disease by either laparoscopy or by robotic or laparoscopic-assisted vaginal hysterectomy from November 2012 to December 2020. Data on UMs, isolated tumor cells (ITCs), cytology, lymphovascular space invasion, free cancer cells in fallopian tubes, stage, histology and grade were collected. Primary and secondary outcomes were cancer recurrence and disease-specific death. Kaplan–Meier curves and multivariate logistic regression were used for statistical analysis. Results: A total of 930 women with early-stage endometrial cancer were included; 789 (84.8%) had hysterectomy with a uterine manipulator and 141 (15.2%) without. A total of 88% had endometrioid histology, 71.6% were grade 1 and 95.7% had stage I disease. A higher risk of recurrence was observed with the Hohl manipulator (HR: 2.83. 95% CI: 1.004–7.98 *p* = 0.0492) on univariate analysis. On multivariate analysis, neither UM was associated with recurrence. With a mean follow-up of 48 months (range 3–118), no effect was seen on disease-specific death in either Hohl or V-Care (HR: 1.66. 95% CI: 0.48–5.70 and HR:1.29. 95% CI: 0.33–4.98). In high-grade histologies, UMs were strongly associated with recurrence (HR: 12.1. 95% CI: 1.52–96.6 *p* = 0.019) and disease-specific death (HR: 10.2. 95% CI: 1.12–92.1 *p* = 0.032). Conclusions: The use of UMs in MIS for endometrial cancer was associated with higher rates of recurrence without affecting disease-specific death, except in high-grade histologies.

## Simple Summary

Endometrial cancer is the most common gynecologic cancer worldwide and is usually primarily treated by minimally invasive surgery (MIS). Uterine manipulators help surgeons throughout dissection and facilitate hysterectomy. In cervical cancer, there is evidence that uterine manipulator use in MIS increases the risk of recurrence and mortality, and uterine manipulator use was identified as a possible risk factor. Concerns were then raised regarding the oncological safety of uterine manipulators in endometrial cancer surgery, which could result in higher recurrence rates and mortality.

## 1. Introduction

Endometrial cancer (EC) is the most frequent gynecological cancer worldwide, with more than 417,000 patients newly diagnosed in 2020 [1]. The majority of endometrial cancers are primarily treated with surgery. Minimally invasive surgery (MIS) such as robotic, laparoscopic or laparoscopically assisted vaginal hysterectomy are commonly used to minimize perioperative morbidity and mortality. Uterine manipulators (UMs) have been used in benign gynecologic surgeries in order to help surgeons throughout the pelvic dissection, uterine mobilization and colpotomy [2].

Recently, Ramirez et al. compared MIS with laparotomy in the surgical management of early-stage cervical cancer and showed more recurrences and deaths in the MIS group (LACC trial) [3]. Many hypotheses have been raised to explain these worse outcomes, including the use of UMs [4]. In early-stage endometrial cancer, recent publications have shown worse oncological outcomes with UMs. Padilla et al. [5] reported a higher risk of recurrence with UMs (11.69% vs. 7.4%, *p* < 0.001) and a higher risk of death (HR 1.74; 95% IC 1.07–2.83). The use of UMs and their contact with tumor tissue during surgery may worsen the outcome, but this mechanism is not totally understood. The risks of uterine perforation, peritoneal spilling, myometrial trauma and cervical laceration are unique to the use of UMs. On the other hand, Uccella et al. reported a similar rate of recurrence regardless of UM use (13.5 vs. 11.6%), without significant differences in terms of disease-specific death [6].

The National Comprehensive Cancer Network recommends the MIS approach for endometrial cancer surgery, but the issue of UMs is not discussed [7]. This is of concern since the safety of UMs has not been thoroughly investigated and may be associated with poorer outcomes with potential tumor spreading and higher recurrence rates [5]. Therefore, the safety of UMs in EC needs to be further assessed.

The aim of our study was thus to assess the oncological safety of uterine manipulators in early-stage endometrial cancer treated by MIS.

## 2. Materials and Methods

This is a retrospective single center cohort study of patients with early-stage endometrial cancer who underwent MIS with or without UMs. In accordance with the journal’s guidelines, we will provide our data for independent analysis by a team selected by the Editorial Team for the purposes of additional data analysis or for the reproducibility of this study in other centers, if this is requested.

Inclusion criteria included patients with epithelial stage I and II (FIGO 2009) [8] who underwent total hysterectomy, bilateral salpingo-oophorectomy and lymph node evaluation (indocyanine green sentinel node biopsy with or without pelvic lymphadenectomy) between November 2012 and December 2020. Patients with no residual disease on pathology report were included since recurrence may still occur in this group of patients. Exclusion criteria included patients with non-epithelial endometrial neoplasia, advanced-stage disease (FIGO stage III–IV) or atypical endometrial hyperplasia.

All patients underwent minimally invasive surgery performed by the robotic, laparoscopy or LAVH approach depending on patient characteristics. Patients’ characteristics and follow-up information were extracted retrospectively from medical records. The following surgical and histopathological variables were collected: type of uterine manipulator used, prior tubal ligation, histology, grade, stage, myometrial infiltration, lymphovascular space invasion (LVSI), isolated tumor cells (ITC), peritoneal washings and free cancer cells in fallopian tubes (floaters). Information on adjuvant treatment was also collected (brachytherapy, external beam radiotherapy (EBRT) and chemotherapy). Diagnosis of recurrence was confirmed by either imaging or pathological findings during follow-up.

The primary outcome was cancer recurrence and the secondary outcome was disease-specific death. Data was analyzed using mean standard deviation for numeric variables. Cox regression for univariate and multivariate analysis was used to assess the effect of uterine manipulators. A 95% confidence interval was fixed for all statistical analyses. *p* values were calculated based on the chi squared or Pearson T-test. This study has received our institution’s IRB approval.

## 3. Results

**Figure 1:**
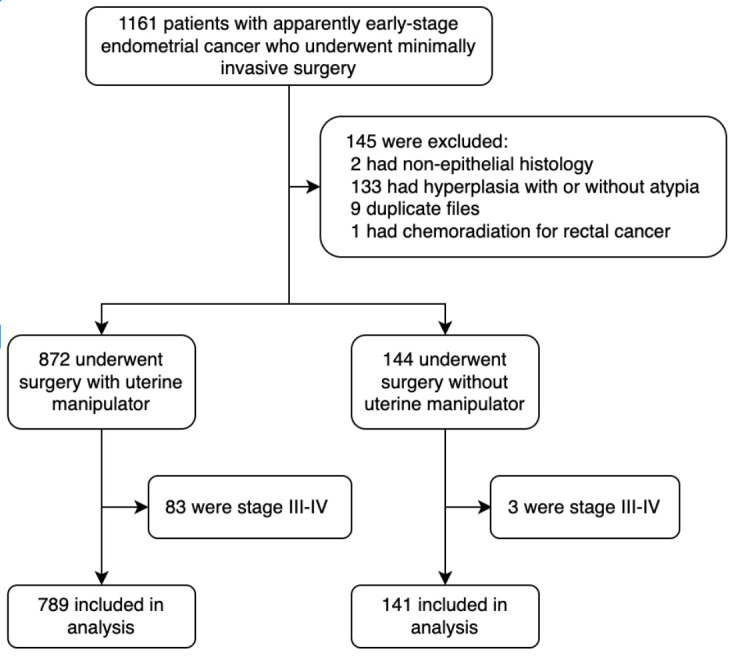
Flow chart.

From November 2012 to December 2020, we identified 1161 women with endometrial cancer at our institution. A total of 930 patients meeting the study criteria were included in the analysis (flow chart, Figure 1). Patients were divided into three groups: no uterine manipulator used (141, 15.2%), V-Care manipulator (267, 28.7%) and Hohl manipulator (522, 56.1%). Altogether, a uterine manipulator was used in 789 patients (84.8%) (Table 1).

Patients had a serous (5.3%), mixed (2.8%), carcinosarcoma (2.1%), clear cell (0.1%) or dedifferentiated (0.06%) histology and had mostly grade 1 (71.6%), stage I (97.1%) disease (Table 2). There was no statistical difference between the three groups with respect to FIGO stage, myometrial infiltration, LVSI, positive peritoneal cytology, prior tubal ligation or presence of ITCs. However, high-grade histologies were more frequent in the UM group (*p* = 0.03), mainly the serous type. There were statistically more fallopian tube floaters in the V-Care UM group (*p* < 0.0001). The rate of adjuvant treatment was similar among the three groups (Table 2).

**Figure 2:**
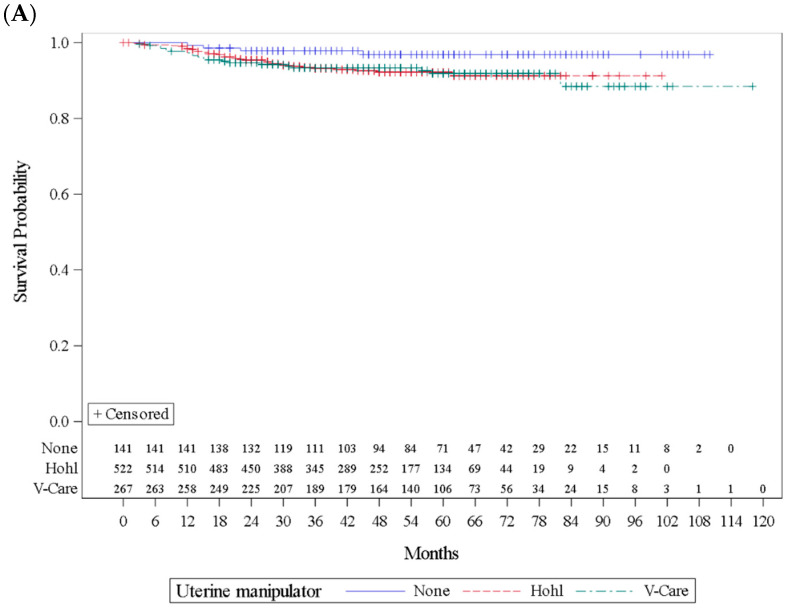

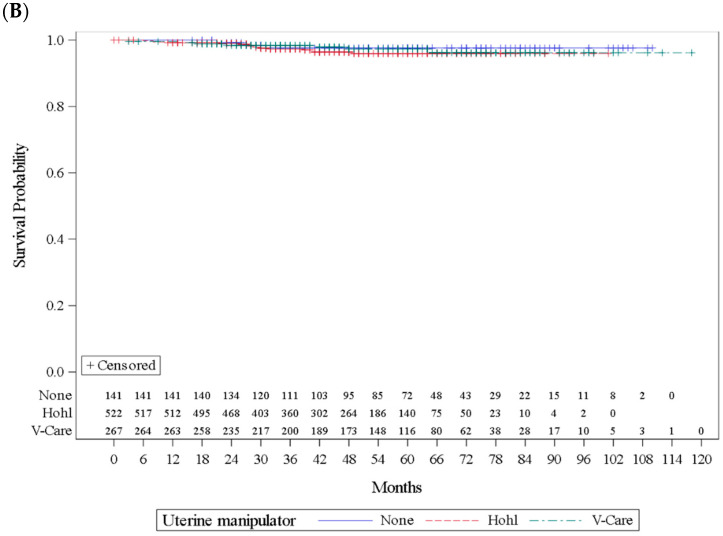
Kaplan–Meier curve—entire cohort. Recurrence-free survival HRs for the Hohl and the V-Care manipulator were, respectively, 2.83; 95% CI: 1.004–7.98; *p* = 0.0492; and HR: 2.86; 95% CI: 0.98–8.37; *p* = 0.0551. (**A**). Disease-specific death HRs for the Hohl and the V-Care manipulator were, respectively, 1.66; 95% CI: 0.48–5.70; *p* = 0.423; and HR: 1.29; 95% CI: 0.33–4.98; *p* = 0.715. (**B**). Legend: HR, hazard ratio. CI, confidence interval.

Recurrence rates for the no UM, Hohl and V-Care groups were 2.8 vs. 6.9 vs. 7.5% (*p* = 0.1516) (Table 3). On univariate analysis, a higher risk of recurrence was observed with the use of the Hohl manipulator (HR: 2.83; 95% CI: 1.004–7.98; *p* = 0.0492) while V-Care was not statistically significant (HR: 2.86; 95% CI: 0.98–8.37; *p* = 0.0551) (Figure 2). The most common sites of recurrence were vaginal, distant metastasis and peritoneal carcinomatosis. No effect was seen on disease-specific death in either the Hohl or V-Care group (HR: 1.66; 95% CI: 0.48–5.70; *p* = 0.423; HR: 1.29; 95% CI: 0.33–4.98; *p* = 0.715). The median follow-up was 48 months (3–118 months). Disease-specific death rates were similar on univariate analysis between the three groups (2.1 vs. 3.1 vs. 2.6%) (Table 3).

On multivariate analysis, neither the Hohl nor the V-CARE device was associated with recurrence (Table 4). However, LVSI (*p* = 0.0163), myometrial infiltration (*p* = 0.0111) and high-grade histology (*p* = 0.0186) were statistically associated with recurrence (Table 4). When grouping all high-grade histologies, UMs were strongly associated with recurrence (HR: 12.1; 95% CI: 1.52–96.6; *p* = 0.0186) and disease-specific death (HR: 10.2; 95% CI: 1.12–92.1; *p* = 0.032) (Table 5). Adjuvant chemotherapy was found to be protective for cancer recurrence (HR: 0.29; 95% CI: 0.10–0.82; *p* = 0.0194) (Table 4).

## 4. Discussion

Our data indicate that the use of UMs in MIS for endometrial cancer increases the risk of recurrence without affecting survival on univariate analysis. However, the presence of LVSI, deep myometrial infiltration and high-grade histologies was associated with recurrence. The most common sites of recurrence were vaginal, peritoneal carcinomatosis and distant metastasis. When grouping high-grade histologies together, we observed higher rates of recurrence and disease-specific death on multivariate analysis with the use of uterine manipulator.

This is a retrospective cohort study of 930 early-stage endometrial cancer patients who underwent minimally invasive surgery with either the V-Care or Hohl manipulator compared to a group of patients who underwent laparoscopically assisted vaginal hysterectomy used as the control group (no uterine manipulator). Our results indicate that the use of UMs in low-risk disease does not increase the risk of recurrence and disease-specific death in multivariate analysis. UM use in high-grade histologies strongly increases the risk of recurrence and disease-specific death. Our results contrast with those of Padilla et al., who recently reported a higher risk of recurrence with UMs (HR: 2.31; 95% CI: 1.27–4.20; *p* = 0.006) [5]. However, our recurrence rate was lower overall. Recurrences may occur with MIS even without UM use in low-risk disease [9].

LVSI is associated with recurrence in our population [10]. However, our data do not demonstrate an association between LVSI and the use of UMs (Table 2). A recent study by Sciutiero et al. [11] published a meta-analysis assessing UMs in relation to LVSI, recurrence rates and positive cytology. They reported no increase in the rates of LVSI with the use of UMs, which supports our data. However, UM type was not mentioned in several studies, such that results may or may not differ according to the use of an intrauterine balloon manipulator. In our study, the Hohl manipulator seemed to increase the risk of recurrence on univariate analysis.

Conversely, a more recent multicentric retrospective study by Quintana-Bertó et al. reported higher rates of LVSI with UM use, especially with intrauterine balloon manipulators [12].

Uterine manipulators, particularly ones with an intrauterine balloon, may “push” cells into the myometrium and lymphatic spaces. In addition, UMs may interfere with pathological reports, with artifacts from crush injuries caused by UM installation and intraoperative manipulation. Indeed, nuclear crush injuries on final pathology, endometrial lining disruption and intratubal contaminants following the use of UMs have been reported [13]. In addition, with the Hohl manipulator, the intracervical screw can damage the cervical stroma and tumor architecture in case of cervical cancer involvement or isthmic tumors and compromise the accuracy of the pathological report.

Walker et al. (LAP2 trial) reported a similar recurrence rate between laparoscopy and laparotomy, with a 3-year recurrence rate of 11.4% and 10.2%. Patterns of recurrence were similar between the groups (*p* = 0.470); however, the issue of UMs was not assessed in that study [14]. Janda et al. (LACE trial) confirmed the safety of the laparoscopic approach in endometrial cancer, with similar recurrence rates between the two approaches [15]. However, the use of UMs was not their main outcome, but they used McCartney Tube (OR Company), which does not require intrauterine placement. UM use was not included as a potential risk factor for recurrence. A five-year retrospective study from our center published by Renaud et al. [16] showed a similar recurrence rate between MIS and laparotomy within a similar cohort of patients. Also, the risk of uterine perforation and vaginal uterine extraction unique to MIS may also increase the risk of recurrence; these sometimes require contained uterine morcellation, which may contribute to tumor spreading and worse outcomes. This risk is not seen in open surgery.

Feigenberg et al. reported a uterine weight >75th percentile as a risk factor, with a 2.2 times greater risk of intra-abdominal recurrence in high-grade endometrial cancer [17]. Uncontained uterine vaginal extraction may be associated with fallopian tube or tumor spillage due to the pressure on the uterine corpus. Uterine manipulators were also associated with a 4.2 times greater risk of single-site abdominal recurrence in this population [17].

Even if high-grade histologies are strongly associated with recurrence, it should be noted that the majority of the recurrences in our series occurred in low-risk disease (stage IA, grade 1–2, endometrioid), which is concerning. This result highlights the potential negative impact of surgical technique (uterine manipulation, cavity effraction, uterine extraction) on oncological outcome.

The main strength of our study is the large population of early-stage endometrial cancer (n = 930), with a median follow-up of 48 months (3–118). This is a single-site study with standardized pathology reports with assessment of LVSI. Surgical staging was also thoroughly performed and comparable amongst surgeons.

One of the weaknesses of our study is its retrospective nature and the fact that patients were not randomized according to patient characteristics and histopathological factors. Some surgical details were not always available, such as fallopian tube occlusion prior to uterine manipulation in the case of no prior tubal ligation. The proportion of patients with LAVH (no UM) was low compared to the other two UM groups, which could potentially increase the impact of UMs on oncological outcome. Also, the number of patients with a high-grade histology was very low in the LAVH group, which can also overestimate the impact of UMs.

Without prospective randomized trials, the impact of UMs on endometrial cancer remains controversial. Thus, meticulous intraoperative techniques and surgeons’ experience are key to safe oncological results regardless of the surgical approach. As in cervical cancer, intraoperative contamination of the peritoneal cavity may be an explanation for the rates of subsequent peritoneal carcinomatosis and vaginal recurrence. UMs may also spread tumor cells through the fallopian tubes in patients with no prior tubal ligation and also lead to tumor dissemination, especially with the V-Care, for which we found the highest rate of fallopian tube floaters in our study. Lastly, the risk of uterine perforation and unprotected vaginal extraction of the uterus unique to MIS may also increase risk of recurrence. Since the majority of recurrences in our study occurred in patients with low-risk disease, special attention must be paid to avoid any effraction of the uterine cavity and minimize uterine manipulation throughout surgery.

## 5. Conclusions

In conclusion, our data shows that the use of UMs in MIS for endometrial cancer remains controversial, with higher rates of recurrence without affecting disease-specific death. High-grade tumors have an increased risk of recurrence and disease-specific death with UMs. Many other factors also have to be taken into consideration, such as the role of the pneumoperitoneum, surgical spilling, uterine weight, tubal occlusion, floaters and other intraoperative factors. Surgical techniques should not compromise outcomes, particularly in early-stage low-risk disease. More prospective data with larger cohorts of patients are needed to clarify the impact and safety of UMs in endometrial cancer surgery. The recently launched prospective MANEC trial should provide evidence regarding this issue [18].

## Figures and Tables

**Table 1:** Uterine manipulator use.

Frequency Table			
Variable	Uterine Manipulator	n	% *
Uterine manipulator	None V-Care	141 267	15.2 28.7
	Hohl	522	56.1

% * Percentage after excluding missing values.

**Table 2:** Tumor histology and adjuvant treatment.

		Uterine Manipulator						
		None		Hohl		V-Care		
Variable	Level	n/N	%	n/N	%	n/N	%	*p*-Value ^1^
FIGO 2009 staging	1A	108/141	76.6	428/522	82.0	211/267	79.0	0.4200
	1B	27/141	19.1	83/522	15.9	46/267	17.2	
	2	6/141	4.3	11/522	2.1	10/267	3.7	
Myometrial infiltration	None	56/141	39.7	233/522	44.6	109/267	40.8	0.5643
	<50	57/141	40.4	203/522	38.9	103/267	38.6	
	≥50	28/141	19.9	86/522	16.5	55/267	20.6	
Histology	Carcinosarcoma	1/141	0.7	17/522	3.3	2/267	0.7	**0.0463** (!)
	Clear cell	2/141	1.4	3/522	0.6	4/267	1.5	
	Dedifferentiated	0/141	0.0	4/522	0.8	2/267	0.7	
	Endometrioid	133/141	94.3	447/522	85.6	240/267	89.9	
	Mixed	2/141	1.4	15/522	2.9	9/267	3.4	
	Serous	3/141	2.1	36/522	6.9	10/267	3.7	
Grade	1	106/141	75.2	367/522	70.3	193/267	72.3	**0.0327** *
	2	24/141	17.0	66/522	12.6	42/267	15.7	
	3	11/141	7.8	89/522	17.0	32/267	12.0	
Lymphovascular space invasion (LVSI)	No	126/141	89.4	435/522	83.3	224/267	83.9	0.2084
	Yes	15/141	10.6	87/522	16.7	43/267	16.1	
Positive peritoneal cytology	No	133/141	94.3	500/522	95.8	254/267	95.1	0.7460
	Yes	8/141	5.7	22/522	4.2	13/267	4.9	
Fallopian tube floaters	No	132/140	94.3	492/522	94.3	227/267	85.0	**<0.0001** ***
	Yes	8/140	5.7	30/522	5.7	40/267	15.0	
Isolated tumor cells (ITCs)	No	133/140	95.0	505/522	96.7	249/267	93.3	0.0786 ^T^
	Yes	7/140	5.0	17/522	3.3	18/267	6.7	
Prior tubal ligation	No	99/141	70.2	384/522	73.6	190/267	71.2	0.6391
	Yes	42/141	29.8	138/522	26.4	77/267	28.8	
Brachytherapy	No	98/141	69.5	372/522	71.3	192/267	71.9	0.8804
	Yes	43/141	30.5	150/522	28.7	75/267	28.1	
External beam radiation therapy	No	129/141	91.5	493/522	94.4	252/267	94.4	0.4138
	Yes	12/141	8.5	29/522	5.6	15/267	5.6	
Chemotherapy	No	136/141	96.5	492/522	94.3	259/267	97.0	0.1835
	Yes	5/141	3.5	30/522	5.7	8/267	3.0	

^1^ Based on an exact Pearson chi square test or a Pearson chi square test if (!). Use (!) results with caution. *** <0.001; * <0.05; ^T^ <0.15.

**Table 3:** Recurrence and disease-specific death (univariate analysis).

			Uterine Manipulator						
			None		Hohl		V-Care		
Variable		Level	n/N	%	n/N	%	n/N	%	*p*-Value ^1^
Recurrence	Total	No	137/141	97.2	486/522	93.1	247/267	92.5	0.1516
		Yes	4/141	2.8	36/522	6.9	20/267	7.5	
Recurrence site	Vaginal	No	139/141	98.6	514/522	98.5	259/267	97.0	0.3759
		Yes	2/141	1.4	8/522	1.5	8/267	3.0	
	Peritoneal carcinomatosis	No	139/141	98.6	507/522	97.1	262/267	98.1	0.5034
		Yes	2/141	1.4	15/522	2.9	5/267	1.9	
	Trocart	No	141/141	100	518/522	99.2	266/267	99.6	0.5806
		Yes	0/141	0.0	4/522	0.8	1/267	0.4	
	Pelvic	No	141/141	100	513/522	98.3	264/267	98.9	0.2899
		Yes	0/141	0.0	9/522	1.7	3/267	1.1	
	Distant	No	140/141	99.3	510/522	97.7	263/267	98.5	0.3872
		Yes	1/141	0.7	12/522	2.3	4/267	1.5	
Disease-specific death		No	138/141	97.9	506/522	96.9	260/267	97.4	0.8572
		Yes	3/141	2.1	16/522	3.1	7/267	2.6	
Median follow-up (range), months		48 (3–118)	60 (16–110)		48 (3–101)		57 (3–118)		<0.0001

^1^ Based on an exact Pearson chi square test.

**Table 4:** Multivariate Cox regression analysis—recurrence.

Analysis of Maximum Likelihood Estimates—Recurrence					
Parameter		Estimate	Standard Error	Hazard Ratio (95% CI)	*p* Value
**Uterine manipulator**	**Hohl**	0.40	0.55	1.491 (0.51–4.40)	0.4697
	**V-Care**	0.76	0.56	2.139 (0.71–6.45)	0.1769
**FIGO stage**	**1B**	1.81	1.33	6.124 (0.46–82.48)	0.1720
	**2**	−0.24	0.97	0.787 (0.12–5.31)	0.8059
**Myometrial infiltration**	**<50**	1.02	0.40	2.779 (1.26–6.12)	0.0111 *
	**≥** **50**	−0.15	1.30	0.858 (0.07–10.84)	0.9058
**Histology**	**Carcinosarcoma**	3.19	1.12	24.264 (2.72–216.43)	0.0043 *
	**Clear cell**	−11.41	712.44	0.000	0.9872
	**Dedifferentiated**	2.33	1.54	10.304 (0.50–210.78)	0.1298
	**Mixed**	2.28	1.145	9.825 (1.04–92.85)	0.0462 *
	**Serous**	2.67	1.11	14.413 (1.63–127.18)	0.0163 *
	**High-grade (all)**	2.49	1.06	12.1 (1.52–96.6)	0.0186 *
**Grade**	**2**	0.41	0.42	1.507 (0.67–3.41)	0.3238
	**3**	−0.24	1.09	0.786 (0.10–6.59)	0.8241
**Lymphovascular space invasion (LVSI)**		0.80	0.33	2.218 (1.16–4.25)	0.0163 *
**Peritoneal washing**		0.34	0.55	1.399 (0.47–4.12)	0.5432
**Fallopian tube floaters**		0.28	0.42	1.321 (0.58–3.02)	0.5092
**Isolated tumor cells (ITCs)**		−1.56	1.06	0.210 (0.03–1.69)	0.1419
**Prior tubal ligation**		−0.11	0.32	0.893 (0.48–1.67)	0.7227
**Brachytherapy**		−0.50	0.41	0.611 (0.27–1.36)	0.2262
**External beam radiation therapy**		0.13	0.61	1.143 (0.34–3.810)	0.8283
**Chemotherapy**		−1.24	0.53	0.291 (0.10–0.82)	0.0194 *

* <0.05.

**Table 5:** Multivariate Cox regression analysis—disease-specific death.

Analysis of Maximum Likelihood Estimates—Disease-Specific Death					
Parameter		Estimate	Standard Error	Hazard Ratio (95% CI)	*p* Value
**Uterine manipulator**	**Hohl**	−1.44	0.77	0.24 (0.05–1.07)	0.0607
	**V-Care**	−0.80	0.78	0.45 (0.10–2.06)	0.3036
**FIGO stage**	**1B**	0.37	1.74	1.44 (0.05–43.60)	0.8325
	**2**	1.42	1.30	4.13 (0.32–53.24)	0.2770
**Myometrial infiltration**	**<50**	1.12	0.71	3.08 (0.76–12.38)	0.1138
	**≥** **50**	2.18	1.68	8.85 (0.33–239.26)	0.1950
**Histology**	**Carcinosarcoma**	2.86	1.18	17.50 (1.74–175.94)	0.0151
	**Clear cell**	−15.55	59	0.00	0.9979
	**Dedifferentiated**	2.76	1.67	15.75 (0.59–417.47)	0.0993
	**Mixed**	2.23	1.24	9.33 (0.82–106.34)	0.0720
	**Serous**	2.06	1.22	7.87 (0.71–86.55)	0.0919
	**High-grade (all)**	2.32	1.12	10.16 (1.12–92.1)	0.032 *
**Grade**	**2**	0.54	0.92	1.71 (0.28–10.32)	0.5571
	**3**	2.30	1.22	9.95 (0.91–108.73)	0.0596
**LVSI**		0.92	0.53	2.51 (0.89–7.04)	0.0816
**Cytology**		−14.88	103	0.00	0.9885
**Fallopian tube floaters**		0.50	0.77	1.66 (0.36–7.53)	0.5146
**ITCs**		−0.46	1.21	0.63 (0.06–6.81)	0.7072
**Prior tubal ligation**		0.61	0.50	1.85 (0.69–4.95)	0.2231
**Brachytherapy**		−1.14	0.59	0.32 (0.1–1.02)	0.0543
**EBRT**		−1.06	0.95	0.35 (0.05–2.22)	0.2625
**Chemotherapy**		−1.28	0.69	0.28 (0.07–1.06)	0.0618

* <0.05.

## Data Availability

The original contributions presented in this study are included in the article. Further inquiries can be directed to the corresponding author.
